# Physiological and Psychological Responses during Exercise and Recovery in a Cold Environment Is Gender-Related Rather Than Fabric-Related

**DOI:** 10.3389/fpsyg.2017.01344

**Published:** 2017-08-07

**Authors:** Margarita Cernych, Neringa Baranauskiene, Nerijus Eimantas, Sigitas Kamandulis, Laura Daniuseviciute, Marius Brazaitis

**Affiliations:** ^1^Institute of Sport Science and Innovations, Lithuanian Sports University Kaunas, Lithuania; ^2^Faculty of Social Sciences, Arts and Humanities, Kaunas University of Technology Kaunas, Lithuania

**Keywords:** polyester, merino wool, undergarment, hyperthermia, physiological strain index, psychological stress

## Abstract

We evaluated gender-specific effects of two types of undergarments on exercise-induced physiological and psychological stress and subsequent recovery in cold conditions for male and female participants. Ten healthy men and eleven healthy women (25.0 ± 1.5 versus 23.4 ± 1.2 years old, respectively) completed the experimental session twice with two different types of undergarments: polyester or merino wool leggings and long-sleeve tops; specifically, merino fabric had greater thermal resistance and water absorbency, and less water vapor as well as air permeability than polyester. Experimental sessions involved performing 1 h of exercise on a cycle ergometer at 8°C ambient temperature and 55% relative humidity, holding at 70–80 revolutions per minute and 60% of each participant’s predetermined maximal power output (assessed by maximal oxygen uptake test), followed by 1 h recovery in the same environment. Every 5 min during exercise and every 10 min during recovery, rectal temperature, heart rate, subjective ratings for thermal, shivering/sweating and clothing wetness sensations, and clothing next-to-skin and outer side surface temperature and humidity on the chest, back and thigh were recorded. All participants experienced high physiological stress (assessed by physiological strain index) during exercise. No significant gender differences were found in core temperature or heart rate changes during exercise, but women cooled down faster during recovery. Next-to-skin humidity was similar between genders and different garment sets during exercise and recovery, but such temperatures at the chest during exercise and at the thigh during exercise and recovery were lower in women with both sets of garments. Subjective thermal sensations were similar in all cases. In the last 20 min of cycling, women started to feel wetter than men (*P* < 0.05) for both garment sets. Shivering was reported as stronger in women in the last 10 min of recovery. Most of the changes in the garment microclimates during exercise and recovery in the cold were associated with gender-related differences rather than with fabric-related differences.

## Introduction

While exercising in a cold environment, the elevation of body core temperature (Sawka and Young, 2006) causes an increase in sweating (Sato, 1993). Mainly because of heat transfer by water, which is 25 times greater than by air alone (Toner and McArdle, 2011), heat loss in a cold ambient temperature (T_a_) 5°C has been found to be twice as high when skin and clothing are wet than when dry (Kaufman and Bothe, 1986). Heat production during high-intensity exercise prevents a decrease in core temperature, so warm clothing is unnecessary, but this clothing might not protect the wearer after completion of the exercise, or after being forced to stop exercise because of fatigue or injury (Sawka and Young, 2006). In such health-threatening conditions, an important feature of garments is to evaporate sweat as quickly as possible from the body surface through high water vapor permeability; rather than retaining evaporated sweat, which normally condenses on the outer side of garments with a knitted fabric structure. Garments should also keep the body as warm as possible with low air permeability.

Synthetic garment materials such as polyester (PES) (Gonzalez, 1987) or wool fiber are widely used during physical activity. PES is characterized by poor absorbency, but with organic additives comprising hollow fibers with an inner hydrophobic layer and an outer hydrophilic surface, PES wicks sweat away from the skin better than the more traditional cotton fibers (Watkins, 1984). PES garments resulted in a lower heart rate (HR) and core body rectal temperature (T*_re_*) during 1 h cycling at a T*_a_* of 23°C (Zhang et al., 2001), but during moderate walking and following recovery at a T*_a_* of 2°C, participants felt wetter with PES compared with cotton garments (Ha et al., 1996). In general, wool fibers are both hydrophobic (repelling water) and hygroscopic (absorbing moisture when dry), so they can absorb or give off moisture. They also have better thermal insulating properties than cotton or PES (Holcombe and Hoschke, 1983; Hearle and Morton, 1986). Such properties of wool can help regulate the skin temperature and the microclimate of temperature and relative humidity (RH) in the space between skin and the garment, which in turn can keep the wearer more comfortable under a range of conditions (Li et al., 1992). Among the different wool fibers, merino wool (MER) has been shown to have the greatest amount of crimp and the maximum density of scales, and these two characteristics contribute to its superior thermal insulating capability (Holmér, 1985).

For next-to-skin garments, some physiological benefits (i.e., lower HR and later onset of a sweating response) in wearers have been reported for upper-body single layer MER garments compared with PES garments while exercising. This consisted of 30 min running at 70% of maximal oxygen consumption (VO_2*max*_), 10 min walking at 40% of VO_2*max*_ in a T*_a_* 8°C with an air flow of ∼11 km/h during running and ∼6 km/h during walking (Laing et al., 2008). However, these benefits disappeared when an air flow of ∼7.2 km/h was applied during the same exercise and T*_a_* conditions (MacRae et al., 2014). These two studies revealed no differences for any perception of wearer responses between first-layer garments (MER vs. PES). Exercise-induced analgesia persisting to the recovery period (up to 30 min) (Beaumont and Hughes, 1979) seems likely to have blunted the wearers’ perceptions of any differences between the undergarment types. Moreover, wool can easily absorb up to 30% of its weight in moisture without feeling damp or clammy, and that helps keep a layer of dry air next to the skin which, in turn, helps to hold in body heat. Therefore, the 3–4 g of moisture absorbed by MER and PES garments during exercise (MacRae et al., 2014) might have failed to negate any impact of the clothing type. However, to test this garment-specific response hypothesis, a study involving a longer exercise-induced sweat release and post-exercise recovery in a T*_a_* 8°C (to induce a shift from heat to cold thermogenesis) (Gavit et al., 2001; Brazaitis et al., 2010) is necessary.

In general, manufacturers do not separate sportswear materials for women and men. Men have higher sweating rates (Keatisuwan et al., 1996) and predominantly show evaporative heat loss (Inoue et al., 2005) and commonly have a greater total lean body mass than women (Anderson, 1999). In contrast, women generally have a greater body surface area (BSA)-to-mass ratio and higher percentage of body fat (Anderson, 1999), and lose more heat by convection (Inoue et al., 2005). Nevertheless, in the present study we questioned whether garments from the same sportswear materials and knit structure could be equally physiologically and/or psychologically efficient for male and female subjects while exercising and recovering in the cold. It has been demonstrated that whole body cooling in cold water (2°C) after exercise-induced hyperthermia (T*_re_* 39.5°C) had a greater cooling rate in female than in male subjects (Lemire et al., 2009), but this gender-specific difference disappeared when an acute cold stress was induced by immersion in 14°C water from normothermic conditions (Solianik et al., 2014, 2015). Moreover, women are more sensitive to thermal stimuli and experience greater thermal discomfort related to temperature changes than men (Lautenbacher and Strian, 1991; Golja et al., 2003; Gerrett et al., 2014). Hence, there is a question as to whether whole body cooling after exercise-induced hyperthermia in cold air has a similar gender-specific response on cooling rate to that found in cold water, and whether wearing MER or PES full-length first layer garments can modulate or prevent these health threatening responses.

In this study, we aimed to evaluate gender-specific effects on exercise-induced physiological and psychological stresses followed by recovery in cold ambient conditions in subjects wearing two different types of undergarments. Microclimates created by the different first layer of garment sets were analyzed without adding extra microclimates created by second layers, which could blunt the true response (MacRae et al., 2014). We expected that MER fibers compared with PES fibers might have a greater capacity to absorb moisture and release heat to/from surrounding air or skin, in a temperature- and RH gradient-dependent manner (Shin et al., 2016). We hypothesized that both genders, when carrying out similar intensity exercise regimens, would reach a lower T*_re_* and HR while wearing the MER garment set compared with the PES set because of higher water absorption in the former. The high-intensity exercise used in this study aimed to induce a high level of physiological stress [assessed by physiological strain index (PSI), Moran et al., 1998], which might blunt sensitivity to wearing (Beaumont and Hughes, 1979) the different garment types in both genders. During the post-exercise resting period in T*_a_* 8°C conditions, hyperthermic women might cool down faster than hyperthermic men and feel higher thermal discomfort (Gerrett et al., 2014) and heat loss while wearing the PES garment set compared with the MER set.

## Materials and Methods

### Participants

After being informed of the purpose, experimental procedures and known risks of the study, 10 men and 11 women volunteered and signed a written informed consent to participate to this study. They were considered healthy and physically active with (1) age 20–30 years; (2) homogeneous relative VO_2*max*_ (ml⋅min^-1^⋅kg^-1^); (3) no excessive sport activities, i.e., <3 times per week; (4) no involvement in any temperature-manipulation program or extreme temperature exposure for ≥3 months; (5) non-smokers; and (6) no medications that could affect natural thermoregulation and/or tolerance to fatigue. Subjects with Raynaud’s syndrome, asthma, neurological pathology, or conditions that could be worsened by exposure to cold environment or by high intensity exercise were excluded from this study.

All procedures were approved by LUHS Kaunas Region Biomedical Research Ethics Committee.

### Experimental Design

#### Rationale for the Experiment

The experiment was designed to induce whole-body moderate hyperthermia (T*_re_* ∼38.5°C; Lucas et al., 2015) and heat stress-induced sweat release (0.8–1.4 L/h; Armstrong et al., 1993) in subjects performing sustained high-intensity aerobic exercise (60 min) and thereafter induce a thermoregulatory shift from heat to cold thermogenesis during their post-exercise recovery phase (60 min) in a T*_a_* 8°C, and to investigate the physiological and psychological gender-specific responses in male and female subjects wearing full-length first-layer garments made of MER or PES.

#### Experimental Garments

The physical characteristics of the experimental clothing sets are given in **Table 1**. All garments were made by the same manufacturer (Omniteksas, Raudondvaris, Lithuania). Each subject was provided with two types of garments, consisting of a long-sleeved shirt and full-length leggings. Subjects were asked for their typical garment size, and the sizes for the two garment sets for each subject were the same. Each set was worn only once. For each subject, the order in which the garment sets were tested was randomized. For permeability to air we used the LST EN ISO 9234:1997 testing standard, for water vapor permeability the cup method STP-1:2014, for absorption ISO 18696:2006 and for thermal resistance the LST EN ISO 11092:2015 testing standard. At 24 h before study, all garments were placed at a thermally neutral T*_a_* of 23°C and RH 30–40%.

**Table 1:** Physical characteristics of the experimental clothing fabrics.

Fabric	Rib	Air permeability (mm/s)	Water vapor permeability (g/m^2^ per 24 h)	Water absorbed (%)	Thermal resistance, R*_ct_* (m^2^K/W)
Merino wool (MER)	1 + 1	2411.5	4177	86.2	0.053
Polyester (PES)	1 + 1	2769.1	4439	53.5	0.027


#### Preliminary Procedures

Each participant visited the laboratory three times. The first time was approximately 1 week before the experiment for assessing maximal oxygen uptake (VO_2*max*_) (Stasiule et al., 2014). The load for the experimental protocol was calculated based on this capacity. Increasing ramp cycling load (ICL) was performed on an electronically braked cycle Ergometrics–800S ergometer (Ergo Line, Medical Measurement Systems, Binz, Germany) at a pedal cadence of 70–80 RPM. The test was started by 3 min of baseline pedaling at 20 W and was increased by 2 W every 5 s until the intensity of cycling could not be maintained at the required level for longer than 10 s. The seat and handlebar positions on the cycle ergometer were adjusted for each subject prior to the initial exercise test and maintained in that position for subsequent tests. VO_2*max*_ was assessed using a mobile spirometry system (Oxygen Mobile, Jaeger/VIASYS Healthcare, Hoechberg, Germany). VO_2*max*_ was used to determine the ICL setting for work intensity based on the highest value of VO_2_ reached during 15 s of exercise. The relative VO_2*max*_ was calculated by dividing the absolute VO_2_ per min with the body mass of the subject.

#### Experimental Protocol

Each participant completed the experimental session twice, each time with a different garment set (the order of testing was randomized), but at the same time of the day, and with sessions at least 5 days apart. Participants were asked to avoid strenuous exercise within 40 h before, food within 3 h, and to avoid any eating or drinking during all experiment sessions (from the first to second weighing). First, the subject’s nude body was weighed. After that, the participant inserted the T*_re_* thermocouple by themselves, as mentioned above, dressed in the relevant test garment set and then entered the climatic chamber (Design Environmental Ltd., Gwent, South Wales, United Kingdom). In the climatic chamber, the subject sat at rest for 10 min. Then, all measured parameters (T*_re_*, HR, subjective ratings, and temperature and humidity of the clothing microclimates) were recorded (set as time t_0_). They then underwent 1 h exercise at a T*_a_* of 8°C and RH 55% on a cycle ergometer holding at 70–80 RPM and at 60% of the subject’s predetermined maximal power output. Every 5 min, T*_re_*, HR, subjective ratings, and clothing surface temperature and humidity were recorded. After the test session, each participant had a recovery period. They sat on a stool for 1 h in the same environment as when they performed the exercise. Every 10 min, all measured parameters were recorded. At the end of experiment each subject was weighed in the nude.

### Experimental Measurements

#### Physical Characteristics of the Participants

Each subject’s anthropometric characteristics (Tanita UK Ltd., Philpots Close, United Kingdom, accuracy ± 0.1 kg) were estimated. The subject’s body surface area (BSA, m^2^) was calculating using the following equations: BSA = 128.1 × weight^0.44^ × height^0.60^ (m^2^) for men and BSA = 147.4 × weight^0.47^ × height^0.55^ (m^2^) for women (Tikuisis et al., 2001). The body’s sweat loss was calculated by subtracting the body mass measured after the experiment from that measured before the experiment. Skinfold thickness (in mm) was measured using a skinfold caliper (SH5020, Saehan, Masan, South Korea) at 10 body regions (chin, subscapular, pectoral, suprailiac, midaxillary, abdomen, triceps, anterior thigh, medial collateral ligament and medial calf). Mean skinfold thickness was calculated from these 10 skinfold sites (McArdle et al., 1992).

#### Measurements of Core Body Temperature and Cardiovascular Responses

The T*_re_* was measured using a rectal thermocouple (Rectal Probe, Ellab, Hvidovre, Denmark; accuracy ± 0.01°C) inserted to 12 cm past the anal sphincter. This was inserted by each participant. The HR was measured (S-625X, Polar Electro, Kempele, Finland) throughout the testing and then consecutive 5 s average HR was used for the analysis. T*_re_* and HR values were recorded after 10 min sitting at rest in the climatic chamber (time t_0_) with an T*_a_* of 8°C and RH 55%; every 5 min during the exercise and every 10 min during recovery. To assess physiological heat stress, we calculated the PSI as described by Moran et al. (1998):

$$PSI=5(T_{ret}-T_{re0})\times {\left(39.5-T_{re0}\right)}^{-1}+5({HR}_{t}-{HR}_{0})\times {\left(180-{HR}_{0}\right)}^{-1}$$

The T_*re*0_ and HR_0_ measurements were taken before exercise; T*_ret_* and HR*_t_* measurements were taken after 60 min of exercise. This index was scaled to a range from 1 (no heat stress) to 10 (very high heat stress) within the limits of the following values: 36.5 ≤ T*_re_* ≤ 39.5°C and 60 ≤ HR ≤ 180 beats min^-1^.

#### Subjective Ratings

The method described by Ha et al. (1996) and adapted by Brazaitis et al. (2010) was used to measure subjective ratings for thermal, shivering/sweating and clothing wetness sensations. Thermal sensation ratings ranged from 1 (very cold) to 9 (very hot), with 5 being neutral. Shivering/sweating ratings ranged from 1 (heavily sweating) to 7 (vigorously shivering), with four being neutral. Reported sensations of clothing wetness ranged from 1 (dry) to 6 (dripping wet). During exercise, participants also were asked about perceived exertion using a Borg rating scale (Borg, 1970) ranging from 6 (no exertion at all) to 20 (maximal exertion). Subjective ratings were recorded at the same time points as the T*_re_* and HR measurements.

#### Temperature and Humidity of Clothing Microclimates

The clothing surface temperature and humidity were measured from six points: three between the skin and the experimental clothing (defined as ‘next-to-skin’) and three points from the outer sides of the garments. However, sweat rates on the chest, back and thigh are greater in men than in women (Ichinose-Kuwahara et al., 2010; Gagnon and Kenny, 2012), so thermistors and humidity sensors (HMP-35A, Vaisala, Helsinki, Finland) were placed on the chest, back and one thigh of all participants. The temperature and humidity of the clothing were recorded every 5 min during the exercise, and every 10 min during the recovery period after exercise (at the same time points as T*_re_*, HR and subjective ratings).

### Statistical Analysis

Data was analyzed using IBM SPSS statistics. The data are presented as the mean ± standard error of the mean (SEM). The data were tested for normality of distribution using the Kolmogorov–Smirnov test. Repeated-measures analysis of variance (ANOVA) considering time × garment material (PES vs. MER) × gender was used to analyze the differences in thermophysiological responses of T*_re_*, HR and in fabric properties (microclimate temperature and next-to-skin and outside humidity values). Where a significant main effect was found, a *post hoc* test with Šidák correction was applied to clarify significant differences. Changes during the exercise and recovery were evaluated separately, using the same statistical analysis. Paired-sample Student’s *t*-tests were used to estimate differences in mean PSI and body sweat loss. The non-parametric Wilcoxon signed-rank exact test was used to compare changes in subjective ratings of perceptions (thermal, shivering/sweating and clothing wittedness sensations) and perceived exertion levels. The changes were calculated between genders with the same garment sets and between different garment sets, but for the same gender during exercise and recovery. For all statistical analyses, *P* < 0.05 was considered significant.

## Results

### Physical Characteristics of the Participants

The female group compared with male group had significantly lower height, body mass, body mass index and BSA, but they had a higher body fat percentage and greater BSA-to-mass ratio (*P* < 0.05) (**Table 2**). The groups did not differ in age, mean skinfold thickness and relative VO_2*max*_.

**Table 2:** Physical characteristics of the participants.

	Male (*n* = 10)	Female (*n* = 11)
Age, y	25.0 ± 1.5	23.4 ± 1.2
Height, m	1.84 ± 0.01	1.69 ± 0.02^∗^
Mass, kg	78.67 ± 1.9	60.07 ± 1.7^∗^
Body mass index, kg⋅m^-2^	23.31 ± 0.49	20.96 ± 0.41^∗^
Body fat, %	14.49 ± 0.70	20.02 ± 1.27^∗^
Body surface area, m^2^	2.00 ± 0.03	1.69 ± 0.03^∗^
Surface to mass ratio, cm^2^⋅kg^-1^	254.14 ± 3.17	284.16 ± 3.04^∗^
Mean skinfolds thickness, mm	10.42 ± 0.96	12.33 ± 1.03
VO_2_ max, ml⋅min^-1^⋅kg^-1^	42.26 ± 1.8	39.49 ± 2.5


*Values are shown as the mean ± SEM. ^∗^*P* < 0.05 for women compared with men.*

### Physiological Variables

#### Exercise Intensity

All participants performed exercise at the same relative intensity. The 60% load of maximal power output for women was 138.25 ± 6.15 W; for men, it was 174.55 ± 6.90 W.

#### Rectal Temperatures

**FIGURE 1:**
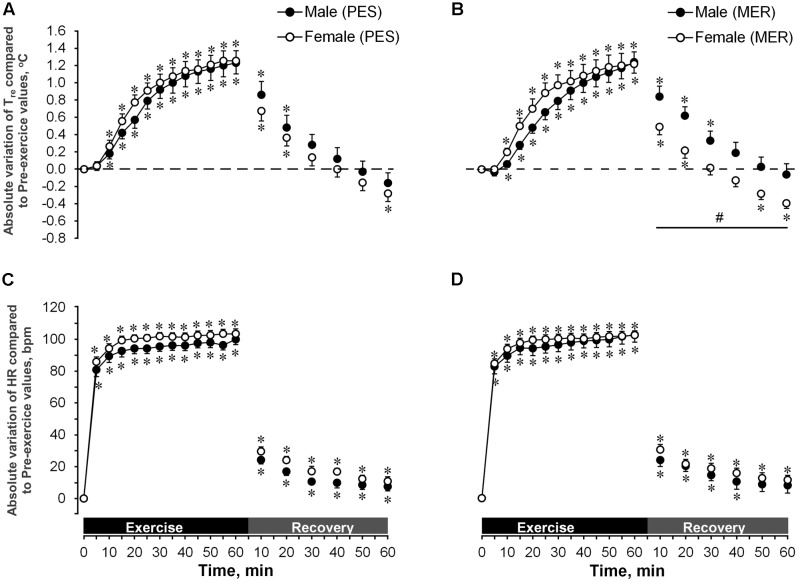
Absolute variation of rectal temperature (T*_re_* in °C) and heart rate (HR in b⋅min^-1^) of individuals wearing polyester (PES) or merino wool (MER) as ‘first layer’ garment sets during exercise and recovery. Pre-exercise value (t_0_) is taken as reference for variation calculation. T*_re_* values in women, 37.4 ± 0.1 for PES **(A)** and 37.5 ± 0.1 for MER **(B)**; in men, 37.2 ± 0.1 for PES **(A)** and 37.1 ± 0.1 for MER (B). HR values in women, 72.2 ± 3.3 for PES **(C)** and 74.3 ± 2.7 for MER **(D)**; in men, 66.0 ± 3.2 for PES **(C)** and 63.9 ± 3.3 for MER **(D)**. Values are shown as the mean ± SEM (10 men, 11 women). ^∗^*P* < 0.05 compared with pre-exercise values; ^#^*P* < 0.05 between genders.

T*_re_* values (all in °C) increased significantly during exercise in all cases (**Figures 1A,B**). Women started performing exercise with significantly higher absolute T*_re_* values (37.4 ± 0.1 for PES and 37.5 ± 0.1 for MER) than men (37.2 ± 0.1 for PES and 37.1 ± 0.1 for MER) and reached significantly higher T*_re_* values at the end of exercise (38.7 ± 0.1 for PES and 38.7 ± 0.1 for MER) compared with men (38.4 ± 0.1 for PES and 38.3 ± 0.1 for MER). Both genders with both sets of garments reached moderate hyperthermic conditions at the end of exercising. There were no significant differences between the two fabrics or between genders for changes in T*_re_* during exercise.

During the recovery phase, men reached their initial T*_re_* faster with PES garments (within 30 min, to 37.4 ± 0.06) than with MER (within 40 min, to 37.2 ± 0.07). The men’s T*_re_* did not fall below their initial temperature during the remaining recovery period. Women cooled down to their initial T*_re_* within 30 min of recovery and, at the end of recovery, their T*_re_* was significantly lower (*P* < 0.05) than their initial T*_re_* value; there was no difference between the PES and MER garment sets. With the MER set, women had significantly lower (*P* < 0.05) T*_re_* values than did men.

#### Heart Rate

Changes in HR during exercise and recovery for the PES and MER garment sets are shown in **Figures 1A,B**. Augmentation of HR (all in beats min^-1^) was significant (*P* < 0.05) during exercise in all cases (PES 99.90 ± 3.31 and MER 102.30 ± 4.62 for men; PES 103.36 ± 3.12 and MER 102.09 ± 2.74 for women), but there were no significant main group effects. The HR recovered to initial values only in men with the MER garments (within 50 min of recovery). In all other cases, the HR stayed significantly higher than the pre-exercise values. Overall, there were no significant differences in the changes in HR between clothing conditions or genders.

#### Physiological Stress

All participants experienced high physiological stress (increased PSI) during exercise (**Table 3**). There were no significant main group effects seen when comparing genders and clothing conditions.

**Table 3:** Physiological strain index (PSI) and relative changes in body mass (sweat loss/BSA).

	PSI	Sweat loss/BSA, kg/m^2^
Male PES	7.21 ± 0.22	0.88 ± 0.09
Male MER	7.08 ± 0.33	0.94 ± 0.08^∗^
Female PES	7.60 ± 0.34	0.89 ± 0.08
Female MER	7.62 ± 0.31	0.96 ± 0.09^∗^


*Values are shown as the mean ± SEM (10 men, 11 women). BSA, body surface area in m^*2*^. ^∗^*P* < 0.05 for wearing different garment fabrics in the same gender.*

#### Sweat Loss

Absolute sweat loss in women was significantly (*P* < 0.05) lower than in men (PES 0.69 ± 0.08 kg and MER 0.74 ± 0.63 kg for men; PES 0.53 ± 0.04 kg and MER 0.57 ± 0.05 kg for women), but the ratio of sweat loss to BSA (**Table 3**) was similar between genders. Both men and women had significantly higher (*P* < 0.05) relative sweat losses when wearing the MER garment sets than with the PES sets.

### Perceptual Variables

#### Perceived Exertion

**FIGURE 2:**
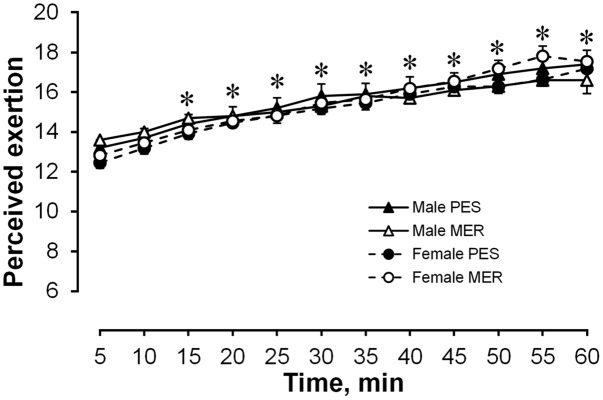
Subjective ratings of perceived exertion. Values are shown as the mean ± SEM (10 men, 11 women). ^∗^*P* < 0.05 compared the fifth and subsequent minutes.

Subjective ratings of perceived exertion increased significantly within 15 min of exercise and by the end of exercise reached a rating of ‘very hard’ (**Figure 2**). No significant main group effects were found.

#### Thermal Sensation

**FIGURE 3:**
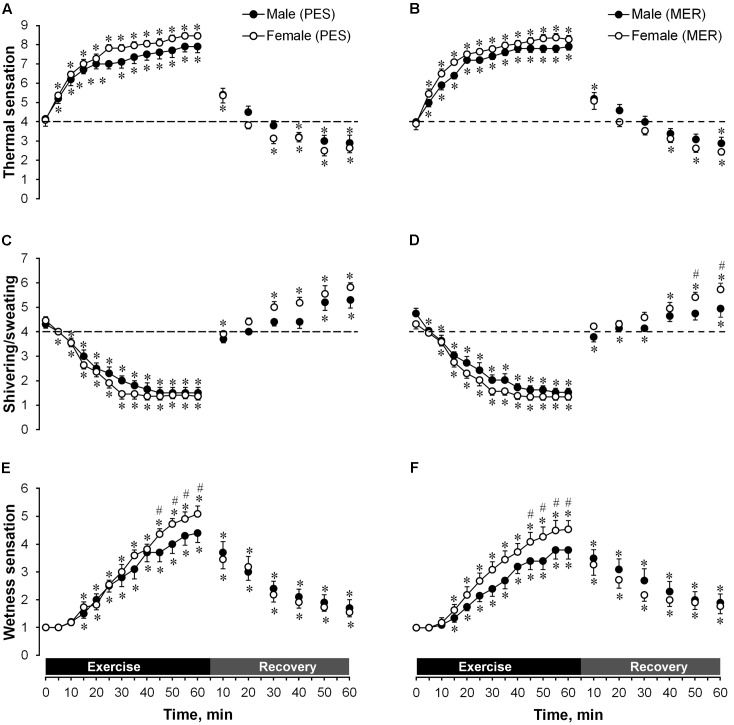
A comparison of temporal changes in thermal sensation wearing **(A)** polyester (PES) or **(B)** merino wool (MER); shivering/sweating sensations wearing **(C)** polyester (PES) and **(D)** merino wool (MER); and wetness sensations wearing **(E)** PES and **(F)** MER garment sets. Values are shown as the mean ± SEM (10 men, 11 women). ^∗^*P* < 0.05 compared with pre-exercise values; ^#^*P* < 0.05 between genders.

**Figures 3A,B** display the responses in thermal sensation during exercise and following recovery in PES and MER clothing conditions for both genders. There was a significant main effect for time (*P* < 0.05), but there were no significant differences between the genders or clothing conditions. Men rated themselves as between ‘warm’ and ‘hot’; women between ‘hot’ and ‘very hot’ in the end of exercise. During recovery, there was a shift from a heat-generated response to cold stress and all participants started to rate themselves as being between ‘cool’ and ‘cold.’

#### Sweating/Shivering Sensation

**Figures 3C,D** display the responses in sweating/shivering sensation during exercise and following recovery in PES and MER clothing conditions for both genders. During exercise there was a significant main effect for time (*P* < 0.05), but there were no significant differences between genders or clothing conditions. All participants rated themselves as being between ‘moderately sweating’ and ‘heavily sweating’ at the end of exercise. During recovery, after shifting from heat to cold stressors, women wearing the MER set reported a significantly higher shivering rating than men with the MER set (between ‘slightly’ and ‘moderately shivering’ vs. between ‘not at all’ and ‘slightly shivering,’ respectively) in the last 10 min of recovery.

#### Wetness Sensation

**Figures 3E,F** display the responses in wetness sensation during exercise and post-exercise recovery in PES and MER clothing conditions for both genders. There was a significant main effect for time (*P* < 0.05), but there were no significant differences between the clothing conditions. For both PES and MER garment sets, wetness sensation was greater in women than in men after 45 min of exercise. At the end of exercise, women rated themselves as between ‘wet’ and ‘dripping wet’ when wearing the PES set, and between ‘sticky’ and ‘wet’ when wearing the MER set; men rated themselves as between ‘sticky’ and ‘wet’ when wearing the PES set, and between ‘damp’ and ‘sticky’ when wearing the MER set.

### Garment Microclimates

#### Humidity Transfer

**FIGURE 4:**
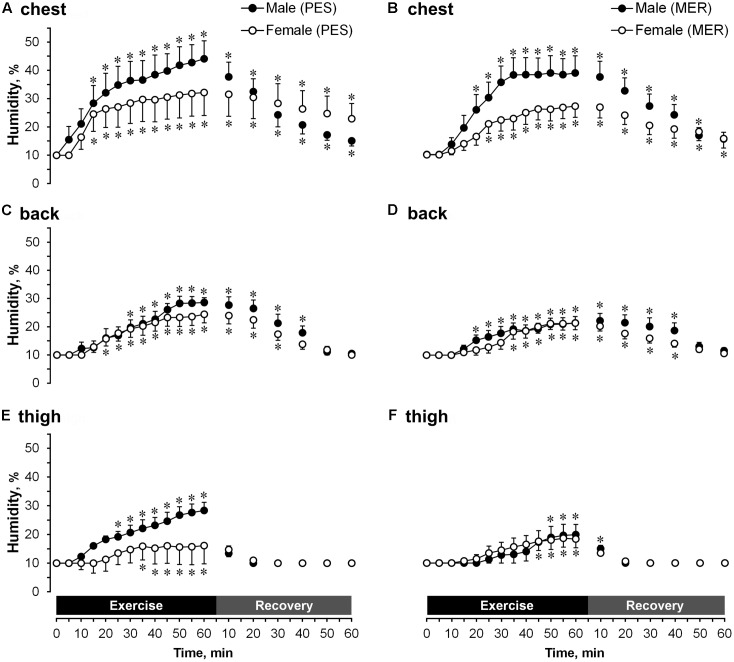
A comparison of temporal changes in garment microclimate humidity next-to-skin values at the chest wearing **(A)** polyester (PES) and **(B)** merino wool (MER) garment sets; at the back wearing **(C)** PES and **(D)** MER garment sets; at the thigh wearing **(E)** PES and **(F)** MER garment sets. Values are shown as the mean ± SEM (10 men, 11 women). ^∗^*P* < 0.05 compared with pre-exercise values.

**FIGURE 5:**
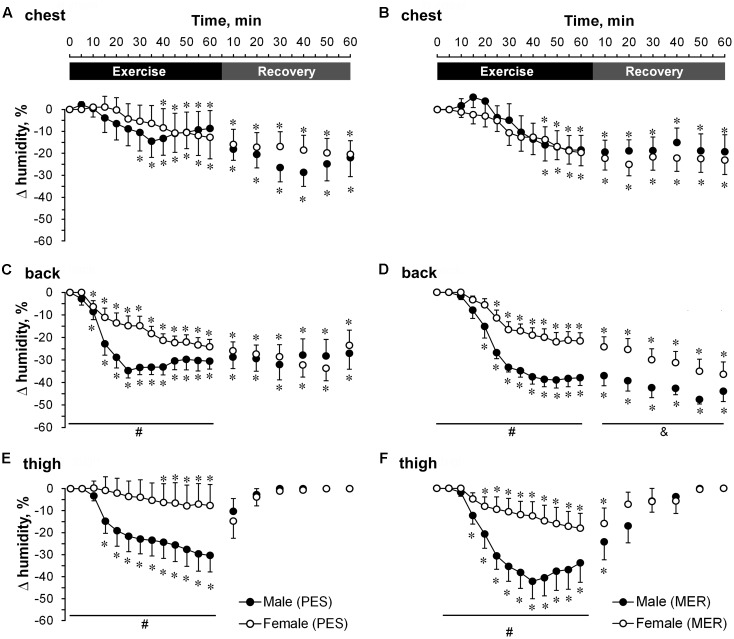
A comparison of temporal changes in garment humidity values at the chest wearing **(A)** polyester (PES) and **(B)** merino wool (MER) garment sets; at the back wearing **(C)** PES and **(D)** MER garment sets; at the thigh wearing **(E)** PES and **(F)** MER garment sets. Results are shown as differences in humidity between next-to-skin values and the outer side of garments. Values are shown as the mean ± SEM (10 men, 11 women). ^∗^*P* < 0.05 compared with pre-exercise values; ^#^*P* < 0.05 between genders; ^&^*P* < 0.05 significant difference between men when wearing different garment fabrics.

There were no differences in next-to-skin humidity between genders or types of garment (**Figure 4**). All changes involved differences in humidity outside the garments. A comparison of temporal changes (**Figure 5**) showed that sweat permeability was significantly higher (*P* < 0.05) in men than in women at the back for both garment sets and at the thigh when wearing the MER set during exercise. Sweat permeability at the back differed significantly (*P* < 0.05) between the PES and MER garment sets in men during recovery. Garment humidity returned to the initial values at the thigh in all cases during the recovery phase, but did not reach the initial values at the chest or back.

#### Temperature

**FIGURE 6:**
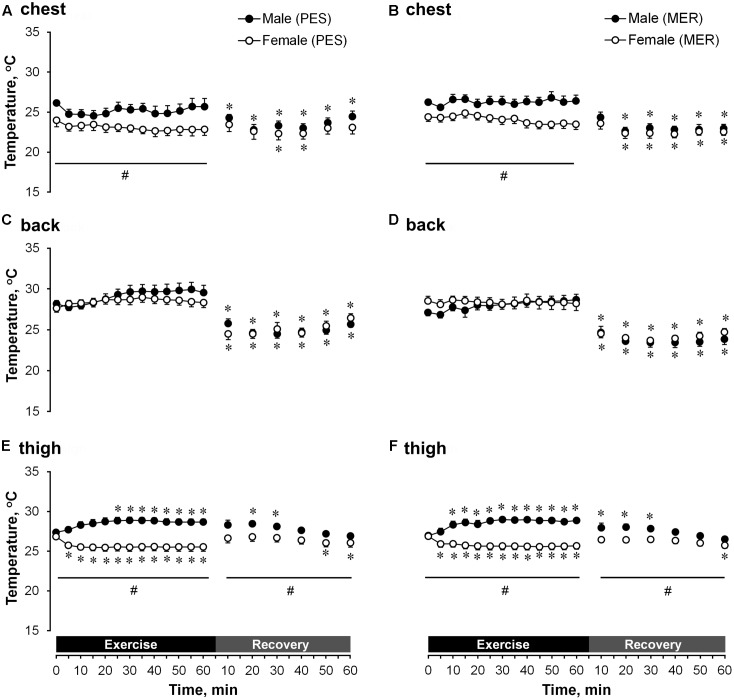
A comparison of temporal changes in garment microclimate next-to-skin temperature at the chest wearing **(A)** polyester (PES) and **(B)** merino wool (MER) garment sets; at the back wearing **(C)** PES and **(D)** MER garment sets; at the thigh wearing **(E)** PES and **(F)** MER garment sets. Values are shown as the mean ± SEM (10 men, 11 women). ^∗^*P* < 0.05 compared with pre-exercise values; ^#^*P* < 0.05 between genders.

**FIGURE 7:**
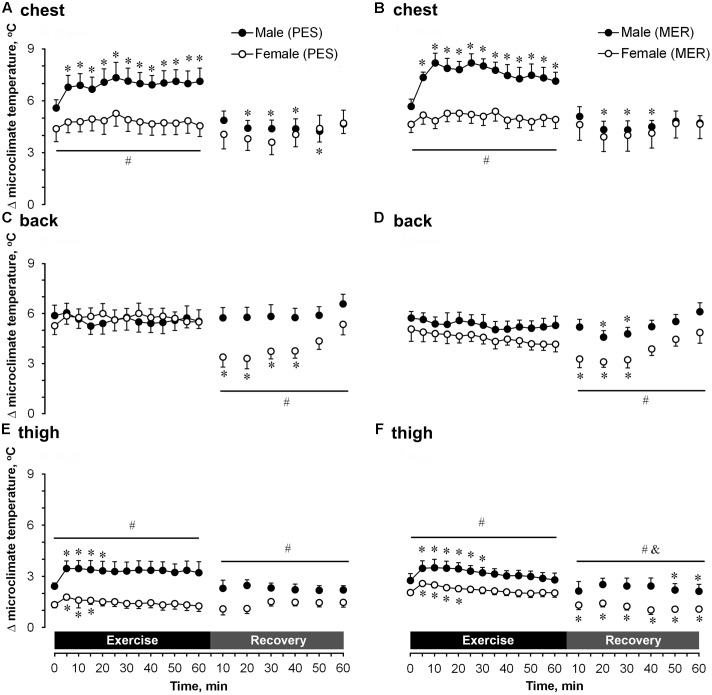
A comparison of temporal changes in garment temperatures at the chest wearing **(A)** polyester (PES) and **(B)** merino wool (MER) garment sets; at the back wearing **(C)** PES) and **(D)** MER garment sets; at the thigh wearing **(E)** PES and **(F)** MER garment sets. Results are shown as differences in temperature between the next-to-skin microclimate and the outer side of garment. Values are shown as the mean ± SEM (10 men, 11 women). ^∗^*P* < 0.05 compared with pre-exercise values; ^#^*P* < 0.05 between genders; ^&^*P* < 0.05 significant difference between women wearing different garment fabrics.

The next-to-skin temperature (**Figure 6**) and differences in temperature between the next-to-skin microclimate and the outer side of garment (**Figure 7**) was significantly (*P* < 0.05) higher in men at the chest and thigh and did not differ at the back (compared with women) during exercise, for both PES and MER garment sets. The next-to-skin temperature was significantly higher (*P* < 0.05) at the thigh in men than in women during recovery for both clothing sets. The differences in temperature between the next-to-skin microclimate and the outer side of garment was significantly (*P* < 0.05) higher in men at chest and thigh (compared with women), and significantly (*P* < 0.05) higher in men at thigh compared PES and MER.

## Discussion

This study aimed to determine whether high-intensity aerobic exercise followed by prolonged recovery (60 min) in a cold environment (8°C) would induce gender-specific physiological or psychological changes and whether wearing MER (natural) vs. PES (synthetic) full-length first layer garments could modulate such responses. In this study, we observed that although both genders experienced similar levels of exercise-induced physiological stresses (increased PSI), which accompanied similar perceived sensations of exertion, women had a greater post-exercise recovery cooling rate (decrease in T*_re_*) than men in cold air. These data agree with those of Lemire et al. (2009) who showed that women had an approximately 1.7-fold greater rectal cooling rate than men when recovering from exercise-induced hyperthermia in cold water (2°C). They also suggested that the BSA-to-mass ratio and body adiposity did not influence core (rectal) cooling rates in previously hyperthermic individuals, and attributed the differences in cooling rates to gender-specific physical differences in lean body mass (Lemire et al., 2009). In our study, men also had significantly higher mean lean body mass (67.3 kg) than women (47.0 kg). Interestingly, there were no gender differences in the rates of core cooling when normothermic participants were immersed in 14°C water (Solianik et al., 2014, 2015), suggesting that differences in lean body mass alone do not fully explain the differences in cooling rates. The greater cooling rates in hypothermic women might be affected by their dominant cutaneous vasodilation heat loss mechanism (Inoue et al., 2005). It is known that women lose more heat by convection than by evaporation, whereas men predominantly show evaporative heat loss (Inoue et al., 2005). Women have a significantly greater BSA-to-mass ratio than men (**Table 2**), and this is one of the indicators for a higher cooling rate (McArdle et al., 1984). Furthermore, it has been demonstrated that temperatures in active and inactive muscles in women stay higher for a longer time after dynamic exercise compared with men (Kenny and Jay, 2007), suggesting a larger blood circulation in skin and muscles recovering from exercise (Halliwill, 2001). Thus, it seems likely that in our study cutaneous vasodilation, heat convection and low ambient temperature played a major role for the greater heat loss seen in hyperthermic women than in hyperthermic men during their recovery in cold conditions.

According to Nielsen and Endrusick (1990) the perception of humidity is mainly correlated with skin dampness. In our study, despite the similar next-to-skin humidity of garments in all measured locations (comparing genders and different fabrics), women felt more wetness than men. Filingeri et al. (2013) showed that humans could mistakenly have a sensation of local skin wetness when in contact with a cold dry surface producing skin cooling rates of 0.14–0.41°C/s. In our case, women had a lower next-to-skin temperature at the chest and thigh during exercise, which could create a sensation of dampness. Humans do not have humidity receptors (Clark and Edholm, 1985) and how we feel ‘wetness’ is still unknown. Ackerley et al. (2012) suggested that humans feel this sensation from complex somatosensory interactions integrating temperature and mechanical inputs at different anatomical levels (Cappe et al., 2009; Ackerley et al., 2012). Wet clothing in a cold environment feels colder then dry clothing. Low temperatures perceived through thermoreceptors such as small myelinated Aδ and unmyelinated C-fibers could play an important role in the perception of local skin wetness (Campero and Bostock, 2010; Filingeri et al., 2013). Wet clothing is also heavier then dry, so it creates higher friction and gives a stronger stimulus to tactile receptors than a dry garment (Nielsen and Endrusick, 1990). Women respond to acute stressors with more intensely negative effects than men because of their greater activity in brain regions that translate stress responses to subjective awareness. This greater activity is found particularly in limbic regions dense with gonadal hormone receptors (Ordaz and Luna, 2012). Therefore, we speculate that the feeling of humidity affects higher central nervous system levels, which are more sensitive in women than in men.

We show that, despite the lower next-to-skin temperature in women, thermal sensation did not differ between genders. We probably feel hot while exercising in the cold by integrating information about increased core temperature, and because the skin receives warmer blood from the body’s center via peripheral vasodilatation. The body core and dermis sense temperature through spino-reticulo-hypothalamic pathways, which project to the preoptic anterior hypothalamus and induce autonomic thermoeffector responses. Two other pathways project to the insula via the spino-thalamo-cortical route and cause thermal and sweating sensations. During post-exercise recovery, when we are sensitive to temperature changes, epidermal thermoreceptors react to decreased heat production in combination with low ambient temperature through the spino-thalamo-cortical pathways, and trigger cold sensations and a shivering response (Romanovsky, 2007; Nakamura and Morrison, 2008). In our study, all participants showed high PSI values during exercise. All stressors affect the activation of specific cognitive processes (Feldman et al., 1995). Thus, exercise-linked stress causes exercise-induced analgesia (EIA) as described by Beaumont and Hughes (1979). They showed that performing exercise with an intensity greater than 50% of VO_2*max*_ for longer than 10 min elicits EIA associated with activation of the endogenous opioid system via neural and hormonal changes during exercise. This has similar effects to morphine and reduces sensitivity to subjective perceptions (Ouzzahra et al., 2012). Therefore, it seems likely that in our study sensitivity to thermal stimuli was suppressed via such EIA and even higher absolute T*_re_* values in women did not influence stronger sensations of warmth. In post-exercise recovery, despite the women’s greater shivering ratings while wearing the MER garment set, subjective ratings of thermal sensations did not differ between genders. Hoffman et al. (2004) showed that EIA-reduced pain ratings persisted into a 30-min recovery phase after exercising at 75% VO_2*max*_ for 30 min. Here, we observed that during post-exercise recovery in a cold environment, heat-induced stressors changed to cold-induced stressors (**Figures 1**, **3**). This suggests that EIA together with a shift from heat-induced thermogenesis to cold-induced thermogenesis had no gender-specific effect on thermal perception during post-exercise recovery in a cold environment.

Proper selection of clothing layers is an important and effective strategy in enabling the wearer to withstand prolonged exposure to cool and cold environmental conditions (MacRae et al., 2014). In general, the effectiveness of clothing as an insulator is mainly because of the entrapment of air (a poor conductor) between the body and garment, within the fabric itself, and bound to body and garment surfaces. There is experimental evidence showing that, compared with synthetic fibers, wool can better mitigate the accumulation of free moisture between fibers and yarns in a fabric (Schneider et al., 1992), and buffer changes in both temperature and humidity under transient conditions (Li et al., 1992). Considering this, in our study we expected that the MER garment set with lower thermal permeability than the PES fabric, together with its higher thermal resistance, and specifically containing both hydrophobic and hygroscopic properties would demonstrate superior microclimate responses for humidity and temperature transfer during exercise and recovery in the cold. However, we found that for most of the variables measured in our conditions both the PES and MER garment sets performed similarly in terms of garment microclimate, and the subjects’ physiological and psychological responses. Moreover, variability in clothing microclimate during exercise and recovery highlights that overall clothing performance is governed by multiple factors in the context of dynamic and realistic wearing conditions. This finding provides further evidence that the performance of clothing systems during exercise is complex, and comparisons based on fiber or fabric characteristics alone are unlikely to reflect this complexity (MacRae et al., 2014). In addition, changes in garment microclimate during exercise and recovery in cold environments had a gender-specific response in our study. Experimental evidence has shown that men have higher local sweat rates on the back, chest (Havenith et al., 2008) and thigh (Inoue et al., 2014) than women. Consistent with this, we observed greater local sweat release in men than women on warmer skin surfaces, which wet both fabrics and then spread out and evaporated. Thus, our results indicate that sweat was properly adjusted by transfer from next-to-skin to the outer side in both garment sets. However, the lack of statistical significance between the two garment types might have been because of insufficient sweat was released and the high variabilities in humidity and temperatures measured within subjects.

Despite the similar microclimates in terms of humidity and temperature, it seems that MER garments, rather than PES fabrics, induced relatively greater sweating in the cold for both genders. It has been suggested that fabrics with poorer absorptive capacity retain moisture on the skin and inhibit further sweat stimulation, thereby reducing fluid losses (Laing et al., 2008). Ha et al. (2010) found a similar tendency in that local sweat rates were greater in cotton clothing with high moisture absorption and high air retention than in polyester clothing with low moisture absorption and high air permeability. Presumably, some sweat was absorbed by the MER fabric in our study because of merino yarn’s ability to absorb liquids, but poor capacity to wick sweat away, as for PES fabrics. MER garments accumulate sweat within the fabric, and in combination with long lasting cutaneous vasodilation after exercise in women, MER rather than PES garments were associated with greater cooling rate and shivering sensations during recovery. According to Moyen et al. (2014), increases in RH also increase sweating rates, so we hypothesize that the MER garments, which cover the body with absorbed sweat, induced an effect similar to a humid environment. Because of that, the relative sweating rates were higher when wearing MER garment sets during exercise compared with the PES sets.

### Limitations

The complexity of garment end performance during wear is often not reflected adequately in comparisons based upon fabric properties alone (MacRae et al., 2014). In agreement with this, we observed high variabilities together with lack of statistical significance between the microclimate of two garment types measured within subjects during wear while exercising and recovering in a cold environment. Conceivably, some of our results might have reached significance with a larger sample size. The present study is not statistically underpowered; that said, power analysis indicated a power of 0.80–1.00 for significant variables with low sample size (10 men and 11 women). Moreover, our environmental conditions were carefully controlled, which makes it difficult to transfer conclusions to real-life varying conditions. Thus, inferences about garment characteristics drawn from fabric properties should be made in cognisance of these limitations. Another point to consider is that reproductive hormones in females may influence the thermoregulatory system (Brooks et al., 1997; Farage et al., 2008), mood (Miller and Miller, 2001), perception (Gescheider et al., 1984), mental and physical function (Farage et al., 2008) during exercise and recovery in the cold. Estrogen and progesterone levels are reported to alter baseline core temperature (Brooks et al., 1997; Farage et al., 2008). In the present study, there were no significant differences in baseline T*_re_* in female subjects between two experimental visits, suggesting similar menstrual cycle condition (i.e., similar hormone level). Moreover, effects of different menstrual cycle phases on investigated physiological and psychological responses in our study were out of scope and thus not evaluated. Another limitation of our study is that weaker thermoregulatory, perception and neuromuscular system response to exercise and temperature conditions in children and older people (Kenney and Munce, 2003; Gorianovas et al., 2013; Brazaitis et al., 2017) suggest that the results of the present study may not be directly applicable to children or older people.

## Conclusion

Despite the similar level of relative loss in body mass, similar sweating sensations and similar next-to-skin humidity values in women and men, women felt wetter at the end of the exercise sessions in cold air in this study. However, the lower next-to-skin temperature in women than in men did not affect thermal sensations differently. Post-exercise recovery in cold air induced a greater T*_re_* cooling rate in hyperthermic women to below the initial level. In contrast, in hyperthermic men the T*_re_* value returned to initial levels for both sets of garments. However, it is remarkable that although the MER garment set induced greater sweating in both genders than did PES garments, the T*_re_* cooling rate and greater shivering sensations were more pronounced in hyperthermic women than in hyperthermic men when wearing the former. Most of the changes in the garment microclimates during exercise and recovery in the cold were associated with gender-related differences rather than with fabric-related differences. This is the first study to find evidence supporting the idea of gender-specific differences for the proper selection of fabrics when exercising and recovering in cold environments, and will be relevant for manufacturers who construct sportswear garments for men and women.

## Author Contributions

The author MB contributed to the design of the work. The authors MC, NB, NE, LD, and MB performed the experiments. The authors MB and MC contributed to the analysis and interpretation of data for the work. The authors MB and MC drafted the work for important intellectual content. The authors MC, NB, NE, LD, SK, and MB finally approved the version to be submitted. The author MB contributed to the revision of this work. All the authors agreed to be accountable for all aspects of the work in ensuring that questions related to the accuracy or integrity of any part of the work are appropriately investigated and resolved.

## Conflict of Interest Statement

The authors declare that the research was conducted in the absence of any commercial or financial relationships that could be construed as a potential conflict of interest.
